# p53-mediated miR-18 repression activates HSF2 for IGF-IIR-dependent myocyte hypertrophy in hypertension-induced heart failure

**DOI:** 10.1038/cddis.2017.320

**Published:** 2017-08-10

**Authors:** Chih-Yang Huang, Pei-Ying Pai, Chia-Hua Kuo, Tsung-Jung Ho, Jing-Ying Lin, Ding-Yu Lin, Fu-Jen Tsai, V Vijaya Padma, Wei-Wen Kuo, Chih-Yang Huang

**Affiliations:** 1 Translation Research Core, China Medical University Hospital, China Medical University, Taichung, Taiwan; 2 Division of Cardiology, China Medical University Hospital, Taichung, Taiwan; 3 Department of Sports Sciences, University of Taipei, Taipei, Taiwan; 4 School of Chinese Medicine, China Medical University, Taichung, Taiwan; 5 Chinese Medicine Department, China Medical University Beigang Hospital, Taiwan; 6 Department of Medical Imaging and Radiological Science, Central Taiwan University of Science and Technology, Taichung, Taiwan; 7 Graduate Institute of Basic Medical Science, China Medical University, Taichung, Taiwan; 8 Department of Biotechnology, Bharathiar University, Coimbatore, India; 9 Department of Biological Science and Technology, China Medical University, Taichung, Taiwan; 10 Department of Health and Nutrition Biotechnology, Asia University, Taichung, Taiwan

## Abstract

Hypertension-induced cardiac hypertrophy and attenuated cardiac function are the major characteristics of early stage heart failure. Cardiomyocyte death in pathological cardiac conditions is the primary cause of heart failure and mortality. Our previous studies found that heat shock factor 1 (HSF1) protected cardiomyocytes from death by suppressing the IGF-IIR signaling pathway, which is critical for hypertensive angiotensin II-induced cardiomyocyte apoptosis. However, the role of heat shock factor 2 (HSF2) in hypertension-induced cardiac hypertrophy is unknown. We identified HSF2 as a miR-18 target for cardiac hypertrophy. p53 activation in angiotensin II (ANG II)-stimulated NRVMs is responsible for miR-18 downregulation both *in vitro* and *in vivo*, which triggers HSF2 expression and the activation of IGF-IIR-induced cardiomyocyte hypertrophy. Finally, we provide genetic evidence that miR-18 is required for cardiomyocyte functions in the heart based on the gene transfer of cardiac-specific miR-18 *via* adenovirus-associated virus 2 (AAV2). Transgenic overexpression of miR-18 in cardiomyocytes is sufficient to protect against dilated cardiomyopathy during hypertension-induced heart failure. Our results demonstrated that the p53-miR-18-HSF2-IGF-IIR axis was a critical regulatory pathway of cardiomyocyte hypertrophy *in vitro* and *in vivo*, suggesting that miR-18 could be a therapeutic target for the control of cardiac functions and the alleviation of cardiomyopathy during hypertension-induced heart failure.

Cardiac hypertrophy is an adaptive response to increase wall stress. In the beginning stages, cardiac hypertrophy has beneficial effects on the maintenance of cardiac output by increasing the size of cardiomyocytes to strengthen contractions in a process called physiological hypertrophy. However, prolonged stress induces systolic dysfunction, leading to pathological hypertrophy and eventually heart failure.

Recent studies have reported that heat shock provided a protective effect during heart failure via the enhanced synthesis of heat shock proteins (HSPs).^1, 2, 3, 4^ The heat shock and stress responses can be modulated by the heat shock transcription factors (HSFs), which specifically recognize the heat shock elements (HSEs) located upstream of target genes. Among the HSFs (HSF1–HSF4), HSF1 and HSF2 are most the studied factors due to their co-expression in most tissues and cell lines.^4, 5^ HSF1 is the main factor responsible for the HSF-HSE signal upon heat shock, whereas HSF2 is more prominently activated during mouse heart development.^6, 7^ Early findings indicated that HSF1 was the cardioprotective factor during heart failure.^8^ However, the role of HSF2 in heart failure has not been extensively investigated.

MicroRNAs (miRNAs) are 21–23 nucleotide long RNA molecules that regulate the stability or translational efficiency of target mRNAs through base pairing with their 3′UTRs.^9^ miRNAs have crucial functions in diverse cellular processes, including cellular differentiation, proliferation, apoptosis and development. Several miRNAs have integrated roles in the regulation of cardiac functions and the progression of heart failure, such as miR204, miR210, miR494, miR15, miR214 and the miR17-92 cluster.^10, 11, 12, 13^ However, relatively little is known about the roles of miRNAs in cardiac injury and their functional consequences in hypertension-induced heart failure.

In this study, we found that HSF2 activated IGF-IIR to induce cardiac hypertrophy during hypertension-induced heart failure. HSF2 expression appeared to be primarily mediated by miR-18, whose expression was severely attenuated by p53 activation in the heart of spontaneously hypertensive rats (SHR). Loss of miR-18 in the heart severely impaired cardiac functions *via* IGF-IIR-mediated cardiac hypertrophy during hypertension. Restoration of cardiac-specific miR-18 expression in spontaneously hypertensive rats alleviated the cardiac defects. Thus, our study uncovered an unanticipated p53-miR-18-HSF2-IGF-IIR pathway in the heart that profoundly influenced cardiac hypertrophy during hypertension-induced heart failure.

## Results

### Recruitment of HSF2 to the *Igf2r* promoter (nt −493 to −463) triggered ANG II-induced IGF-IIR signaling

**Figure 1:**
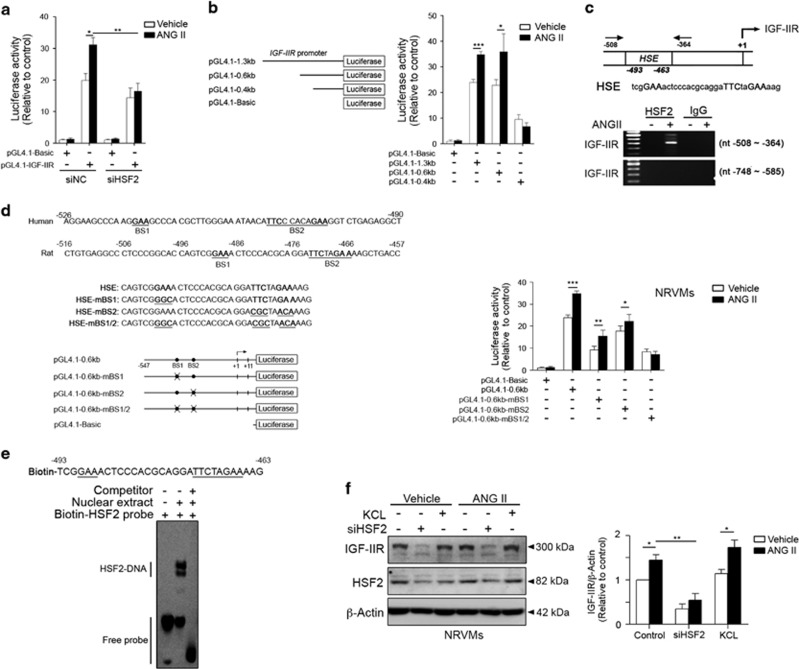
HSF2 was required for ANG II-mediated IGF-IIR expression via transcriptional upregulation. (**a**) NRVMs were simultaneously transfected with the pGL4-IGF-IIR promoter construct (pGF4.1–1.3 kb) and siRNA against HSF2 for 24 h and then treated with 100 nM ANG II for 24 h. The cell lysates were assayed for luciferase activity. Quantification of the results is shown (*n*=3). *P<0.05 and **P<0.01. NC: negative control. (**b**) The schematic diagram of deletion mutations of IGF-IIR promoter constructs. NRVMs were transfected with each IGF-IIR promoter construct for 24 h and then treated with 100 nM ANG II for 24 h. Promoter activity was assessed by luciferase activity. Quantification of the results is shown right (*n*=3). *P<0.05 and ***P<0.001. (**c**) Sequence analysis and transcription factor site prediction identified one putative HSF2 binding element at the IGF-IIR promoter (nt -493 to -463). After treatment with ANG II, the NRVMs were lysed and analyzed by chromatin immunoprecipitation (ChIP). HSF2 binding to the IGF-IIR promoter was quantified using PCR. (**d**) The schematic diagram of point mutations on putative HSEs located on the IGF-IIR promoter. Two HSEs located on the IGF-IIR promoter were mutated to measure its DNA-binding ability (HSE-mBS1, HSE-mBS2 and HSE-mBS1/2). These IGF-IIR promoter construct activities were analyzed by luciferase activity in NRVMs. Quantification of the results is shown right (*n*=3). *P<0.05, **P<0.01 and ***P<0.001. (**e**) The NRVM nuclear fraction was incubated with a biotin-labeled HSF2 probe with the IGF-IIR sequence (nt -493 to -463). After incubation, the mixture was analyzed using an electrophoretic mobility shift assay (EMSA). (**f**) NRVMs were transfected with a siRNA against HSF2 or treated with the HSF2 activator KCL for 24 h and then challenged with 100 nM ANG II for 24 h. The IGF-IIR protein level was detected by immunoblotting. Quantification of the results is shown right (*n*=3). *P<0.05 and **P<0.01. Data represent means±S.D. All presented blots and micrographs are representative of three sets of independent experiments

Our previous studies indicated that HSF1 acted as a repressor to suppress IGF-IIR expression and prevented hypertension-induced heart failure via binding to the heat shock element (HSE) located in the (nt −733 to −706) region of the *Igf2r* promoter.^8^ Intriguingly, another HSE was identified ~0.5 kb upstream of the *Igf2r* open reading frame that was not recognized by HSF1 (Supplementary Figure 1A). We hypothesized that other HSFs participated in the control of *Igf2r* expression. Among the HSFs, HSF1 and HSF2 are abundant in many tissues and cell systems, including the brain and heart. To investigate the influence of HSF2 on IGF-IIR gene expression, we silenced HSF2 expression by using a siRNA targeting HSF2 (Figure 1a). HSF2 knockdown clearly alleviated the ANG II-induced upregulation of *Igf2r* promoter activity (31.18±2.2 *versus* 16.43±2.5-fold change; *P*<0.01) and ANG II-induced target expressions (Supplementary Figure 1B). Moreover, the HIF-1 transcription level, which was negatively regulated by HSF2,^14^ was significantly elevated in HSF2-silenced cells (Supplementary Figure 1B). Luciferase analysis of the generated *Igf2r* promoter deletion segments showed that the *Igf2r*-0.6 kb promoter activities (35.91±7.9 *versus* 22.78±2.23-fold change; *P*<0.001) and *Igf2r*-1.3 kb (34.78±1.21 *versus* 23.89±1.26-fold change; *P*<0.001) were induced by ANG II. However, the *Igf2r-*0.4 kb promoter activity was not stimulated by ANG II, which indicated that the *Igf2r* promoter region between −0.6 kb and −0.4 kb influenced its activities under stress (Figure 1b). Using sequence scanning, we identified a putative conserved HSE located at *Igf2r* (nt −493 to −463) that could be recognized by HSF2 (Figure 1c). To verify that ANG II-induced *Igf2r* upregulation through HSF2, we analyzed the protein–DNA interaction using chromatin immunoprecipitation (ChIP). As shown in Figure 1c, this interaction was only detected at the promoter region (nt −508 to −364) and not at the region (nt −748 to −585) recognized by HSF1. Moreover, this protein–DNA interaction was only detected during ANG II treatment.

Two conserved putative HSF-binding sites (5′-nGAAn-3′) were located in this region (nt −493 to −463) (Figure 1d). To determine which binding site (BS) was involved in the HSF2-mediated *Igf2r* upregulation, the promoter activities of the mBS1, mBS2 and mBS1/BS2 mutants were analyzed. Both mBS1 (~15.50±2.76 *versus* 34.78±1.21-fold change; *P*<0.01) and mBS2 (~22.20±3.11 *versus* 34.78±1.21-fold change; *P*<0.05) partially attenuated the ANG II-induced IGF-IIR promoter activities (Figure 1d). However, mBS1/BS2 significantly abolished the IGF-IIR promoter activities (~7.01±1.59 *versus* 34.78±1.21-fold change; *P*<0.01), suggesting that HSF2 recognition of both BS1 and BS2 was required to influence *Igf2r* expression. In line with these results, the interaction between HSF2 and the *Igf2r* promoter was validated using the biotin-labeled *Igf2r* promoter sequence (nt −493 to −463) via EMSA (Figure 1e). However, the supershift was clearly diminished by a cold probe competitor.

We subsequently activated HSF2 using KCL to observe IGF-IIR expression.^15^ KCL obviously activated HSF2 to increase IGF-IIR expression (Figure 1f), and HSF2 activation markedly augmented ANG II-upregulated IGF-IIR expression. In contrast, HSF2 knockdown significantly alleviated ANG II-induced IGF-IIR upregulation. Taken together, these results demonstrated that HSF2 regulated IGF-IIR expression via recognizing its binding site in the promoter region (nt −493 to −463).

### Nuclear translocation of HSF2 promoted ANG II-induced cardiomyocyte hypertrophy via the IGF-IIR signaling pathway

**Figure 2:**
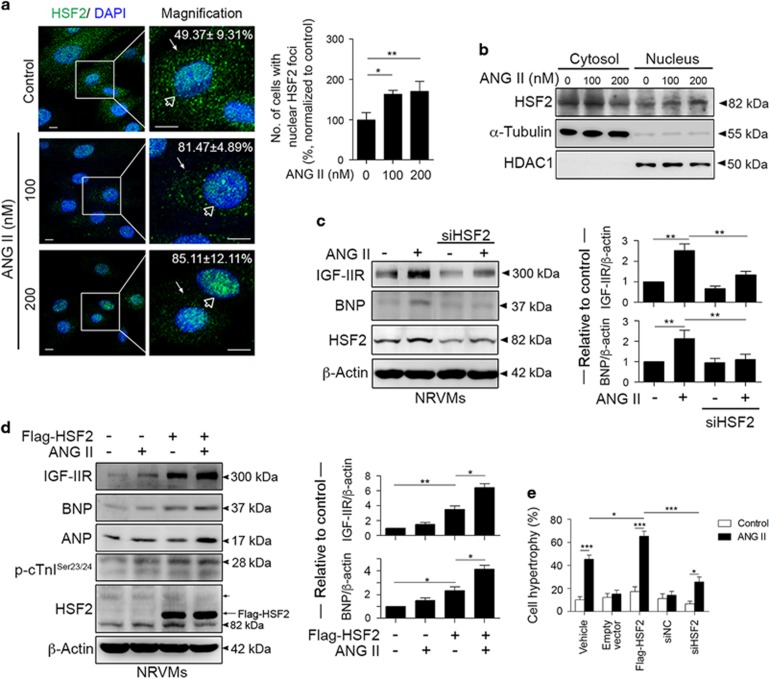
HSF2 nuclear translocation and activation were indispensable for ANG II-mediated IGF-IIR hypertrophic signaling. (**a**) NRVMs were treated with ANG II for 24 h and then the localization of HSF2 was observed by confocal microscopy. A total of 100 cells were counted for the assessment of clear nuclear foci of HSF2. Quantification of the results is shown right (*n*=3). *P<0.05 and **P<0.01. (**b**) H9c2 cells were treated with ANG II for 24 h. The cells were fractionated into the cytosolic and nuclear fraction. (**c**) NRVMs were transfected with a siRNA against HSF2 for 24 h and then treated with ANG II for 24 h. The hypertrophic marker BNP was detected by immunoblotting. Quantification of the results is shown right (*n*=3). **P<0.01. (**d**) NRVMs were transfected with Flag-HSF2 for 24 h and then treated with ANG II for 24 h. The hypertrophic markers BNP, ANP and p-cTnI were detected by immunoblotting. Quantification of the results is shown right (*n*=3). *P<0.05 and **P<0.01. (**e**) NRVMs were transfected with a HSF2 siRNA or Flag-HSF2 for 24 h and then treated with ANG II for 24 h. The cardiomyocyte size was measured using rhodamine phalloidin staining. A total of 100 cells were counted for statistic analysis. Quantification of the results is shown right (*n*=3). *P<0.05, **P<0.01 and ***<0.001. Data represent means±S.D. All presented blots and micrographs are representative of three sets of independent experiments

To confirm that HSF2 translocated into the nucleus to manipulate IGF-IIR expression, we analyzed the distribution of HSF2 after ANG II exposure (Figure 2a). The confocal images showed that cells displaying nuclear HSF2 foci were clearly upregulated following ANG II exposure (81.47±4.89 *versus* 49.37±9.31% *P*<0.01 and 85.11±12.11 *versus* 49.37±9.31% *P*<0.01). The fractionation results showed that the amount of nuclear HSF2 gradually increased (Figure 2b; Supplementary Figure 2A).

Our previous studies demonstrated that activated IGF-IIR recruited Gαq to trigger the intracellular PKCα/CaMKII signaling cascades, thereby contributing to pathological hypertrophy.^16^ Therefore, the expression of the hypertrophic markers atrial natriuretic peptide (ANP), brain natriuretic peptide (BNP) and cardiac-troponin I (cTnI) was assessed. The BNP level was markedly reduced when HSF2 was silenced after ANG II exposure (Figure 2c). However, HSF2 overexpression significantly upregulated ANP and BNP during ANG II exposure. Phosphorylation of cardiac-troponin I (cTnI), which has been reported to increase cTnI phosphorylation by PKC and to contribute to contractile defects in hypertrophy and heart failure,^17^ was also elevated (Figure 2d).

To validate these findings, we transduced H9c2 with lentiviral plasmids carrying a HSF2 shRNA (Supplementary Figure 2B). HSF2 deficiency contributed to reduced IGF-IIR protein expression, which was similar to the results obtained with IGF-IIR^KD^ (Supplementary Figure 2C). Knockdown of HSF2 with the lentiviral shRNA also clearly reduced the expression of the hypertrophic markers ANP, BNP and cTnI (Supplementary Figure 2D). Next, we measured the cardiomyocyte sizes using rhodamine phalloidin staining (Figure 2e). These results revealed that HSF2 upregulated the numbers of cells undergoing ANG II-induced cell hypertrophy (66.42±9.12 *versus* 49.37±9.31% *P*<0.05), whereas knockdown of HSF2 markedly reduced the numbers of cells undergoing ANG II-induced cell hypertrophy (27.13±6.69 *versus* 49.37±9.31% *P*<0.01). Taken together, these results indicated that HSF2 activation contributed to cardiac hypertrophy via IGF-IIR signaling.

### miR-18 downregulation triggered HSF2 expression and IGF-IIR-dependent cardiomyocyte hypertrophy

**Figure 3:**
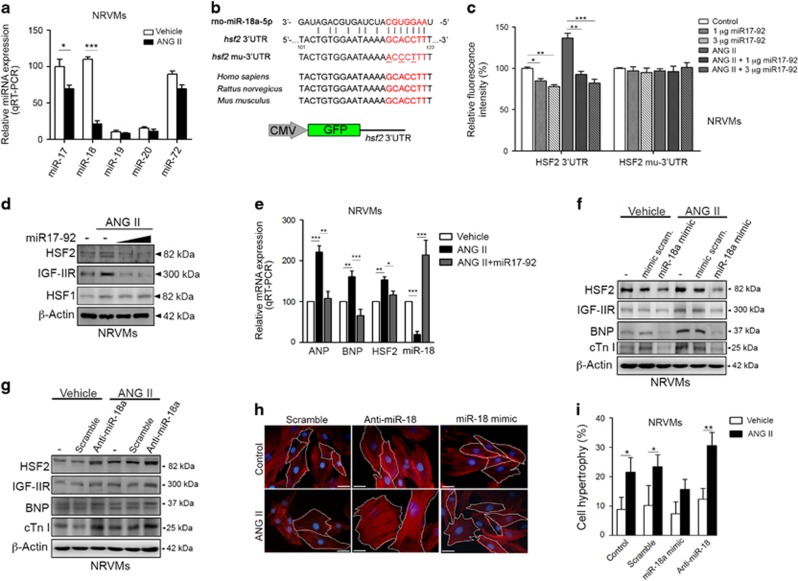
miR-18 downregulation by ANG II resulted in an increase in HSF2 that contributed to IGF-IIR-mediated cardiac hypertrophy. (**a**) NRVMs were treated with ANG II for 24 h. The expression profiles of the miR17-92 cluster were detected by qRT-PCR. Quantification of the results is shown right (*n*=3). ***<0.001. (**b**) Sequence alignment of the miR-18 targeting site in the 3′UTR of human, mouse and rat HSF2. The *hsf2*-3′UTR (~300 bp) is inserted into the pEGFP-C1 vector downstream of GFP. The *hsf2* mu-3′UTR was generated by point mutation in the seed region of the miR-18 targeting sequence. (**c**) NRVM cells were transfected with pcDNA3-miR17~92 and GFP-*hsf2*-3′UTR or GFP-*hsf2*-mu-3′UTR for 24 h. Then, the cells were treated with ANG II for 24 h and the fluorescent intensities were assessed. Quantification of the results is shown right (*n*=3). *P<0.05, **P<0.01 and ***<0.001. (**d**) NRVMs were transfected with 3 μg and 5 μg of pcDNA3-miR17-92 for 24 h and then the cell were treated with ANG II for 24 h. The HSF2 expression level was measured by immunoblotting. (**e**) NRVMs were transfected with pcDNA3-miR17-92 for 24 h and then the cells were treated with ANG II for 24 h. The relative expression levels of ANP, BNP, HSF2 and miR-18 were detected by qRT-PCR. Quantification of the results is shown right (*n*=3). *P<0.05, **P<0.01 and ***<0.001. (**f**) NRVMs were transfected with a scrambled mimic or miR-18 mimic for 24 h and then the cells were treated with ANG II for 24 h. The expression levels of HSF2, IGF-IIR and the hypertrophic markers BNP and cTnI were detected by immunoblotting. (**g**) NRVMs were transfected with a scrambled antagonist or miR-18a antagonist for 24 h and then the cells were treated with ANG II for 24 h. The expression levels of HSF2, IGF-IIR and the hypertrophic markers BNP and cTnI were detected by immunoblotting. (**h**,**i**) NRVMs were transfected with the scrambled, miR-18 mimic or miR-18 antagonist for 24 h and then the cells were treated with ANG II for 24 h. The cardiomyocyte size was measured using rhodamine phalloidin staining. A total of 100 cells were counted for statistical analysis. Quantification of the results is shown right (*n*=3). *P<0.05 and **P<0.01. Data represent means±S.D. All presented blots and micrographs are representative of three sets of independent experiments

Previous studies showed that HSF2 was downregulated by miR-18 through sequence-specific targeting within the 3′-untranslated region (3′-UTR) of the *hsf2* gene transcript during development.^18^ We assessed whether ANG II increased HSF2 expression via miR-18. miR-18 belongs to the miR17-92 cluster, which comprises six miRNAs (miR-17, -18a, -19a, -20a, -19b and -92a) and is transcribed as a polycistron that is subsequently processed to form the individual miRNAs.^9^ Only miR-17 and miR-18 were abundantly detected in NRVMs and H9c2 cells. However, ANG II exposure resulted in severe decreases in these miRNAs (especially miR-18) (Figure 3a; Supplementary Figure 3A). The decrease in miR-18 by ANG II exhibited a dose-dependent response (Supplementary Figure 3B).

To assess whether ANG II negatively regulated miR-18 to elevate HSF2 expression, the GFP intensity of the GFP-*hsf2*-3′UTR containing a miR-18 targeting region was evaluated after miR17-92 cluster overexpression (Figures 3b and c; Supplementary Figure 2C). At baseline, the miR17-92 cluster slightly reduced the GFP intensity of GFP-*hsf2*-3′UTR (84.74±3.00 and 78.08±2.33%) (Figure 3c, left panel). However, overexpression of miR17-92 severely suppressed the ANG II-induced fluorescence intensity in a dose-dependent manner (92.45±4.00 *versus* 136.82±5.74% *P*<0.01 and 82.03±5.01 *versus* 136.82±5.74% *P*<0.001), which clearly indicated the involvement of miR-18 in ANG II-induced HSF2 upregulation. The phenomenon had no significant effect in GFP-*hsf2*-mu-3′UTR-expressing cells, indicating that these targeting regions were critical for miR-18 recognition (Figure 3c, right panel). Moreover, western blotting showed that HSF2 protein expression was downregulated (Figure 3d; Supplementary Figure 3D). These data suggest that endogenous HSF2 is regulated by miR-18 at the mRNA and protein levels. Based on the real-time PCR analysis, miR-18 expression was ~2.2-fold higher after transfection compared with the control (Figure 3e). Additionally, the miR17-92 cluster reduced ANG II-induced upregulation of the cardiac hypertrophy markers BNP (161.33±14.11 *versus* 65.52±16.31% *P* <0.001) and ANP (221.91±15.41 *versus* 107.92±17.32% *P* <0.001), implying that the miR17-92 cluster negatively regulated HSF2 expression to modulate cardiac hypertrophy (Figure 3e). Next, we utilized a miR-18 mimic and inhibitor to monitor ANG II-induced cardiac hypertrophy. Consistently, the miR-18 mimic significantly decreased ANG II-induced IGF-IIR and BNP upregulation via HSF2, whereas the miR-18 inhibitor augmented the effect of ANG II (Figures 3f and g). Moreover, the number of hypertrophic cardiomyocytes was markedly ameliorated in miR-18 mimic-expressing NRVMs under ANG II exposure (15.67±3.41 *versus* 23.43±4.07% *P*<0.05), whereas, miR-18 neutralization by the miR-18 inhibitor significantly upregulated the number of hypertrophic cardiomyocytes under ANG II exposure (30.56±4.53 *versus* 23.43±4.07% *P*<0.05). Taken together, these results demonstrated that ANG II destroyed miR-18 targeting to the miR-18-HSF2-3′UTR to upregulate HSF2 expression, leading to the activation of IGF-IIR-mediated cardiac hypertrophy.

### p53 activation negatively regulated miR-18 to upregulate HSF2

**Figure 4:**
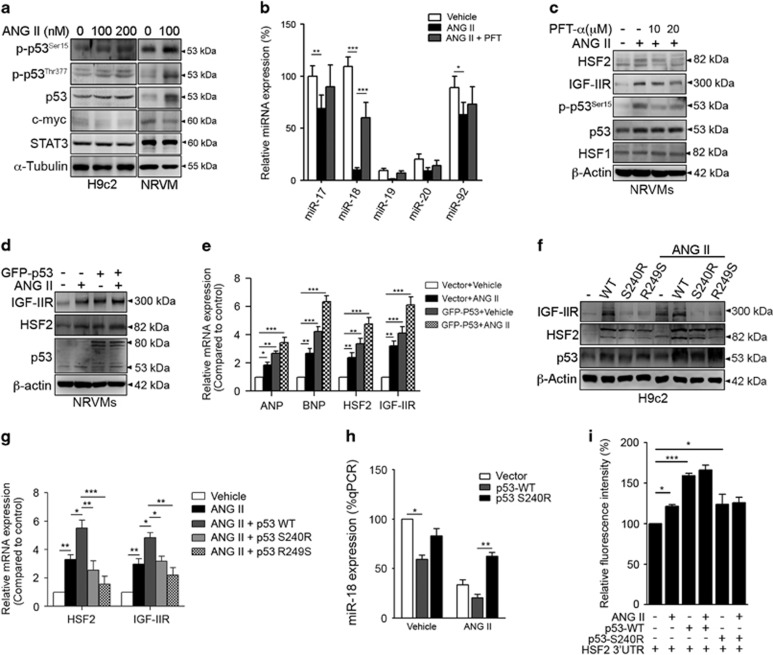
miR-18 is negatively regulated by p53 to manipulate HSF2 expression and thus IGF-IIR-induced cardiac hypertrophy. (**a**) H9c2 cells and NRVMs were treated with various dosages of ANG II for 24 h. The regulatory factors of the miR17-92 cluster were detected by immunoblotting. (**b**) NRVMs were treated with 100 nM ANG II and 10 *μ*M of the p53 inhibitor pifithrin-α (PFT) for 24 h. The miRNA expression levels were analyzed by qRT-PCR. Quantification of the results is shown (*n*=3). *P<0.05, **P<0.01 and ***P<0.001. (**c**) NRVMs were treated with 100 nM ANG II and PFT for 24 h and then analyzed by immunoblotting. (**d**) NRVMs were transfected with GFP-p53 for 24 h and then treated with 100 nM ANG II for 24 h. The cell lysates were analyzed by immunoblotting. (**e**) NRVMs were transfected with GFP-p53 for 24 h and then treated with 100 nM ANG II for 24 h. The relative expression levels of ANP, BNP, HSF2 and IGF-IIR were detected by qRT-PCR. Quantification of the results is shown (*n*=3). *P<0.05 and **P<0.01. (**f**) NRVMs were transfected with p53-WT, p53-S240R (only 50% of the p53 transcriptional activity) and p53-R249S (no p53 transcriptional activity) for 24 h and then treated with 100 nM ANG II for 24 h. (**g**) NRVMs were transfected with p53-WT, p53-S240R and p53-R249S for 24 h and then treated with 100 nM ANG II for 24 h. The relative expression levels of HSF2 and IGF-IIR were detected by qRT-PCR. Quantification of the results is shown (*n*=3). *P<0.05. (**h**) NRVMs were transfected with p53-WT or p53-S240R for 24 h and then treated with 100 nM ANG II for 24 h. miR-18 expression was verified by qRT-PCR. Quantification of the results is shown (*n*=3). *P<0.05 and **P<0.01. (**i**) NRVMs were transfected with GFP-*hsf2*-3’UTR and p53-WT or p53-S240R for 24 h and then treated with 100 nM ANG II for 24 h. The cell lysates were assayed for fluorescent activity. Quantification of the results is shown (*n*=3). *P<0.05 and ***P<0.001. Data represent means±SD. All presented blots and micrographs are representative of three sets of independent experiments

Previous studies demonstrated that several transcription factors directly bound to the miR17-92 promoter, such as stat3,^19, 20^ c-myc,^21, 22^ E2F^23, 24, 25^ and p53.^26, 27^ Therefore, we examined which transcription factor directly influenced miR-18 expression during ANG II treatment. As shown in Figure 3a, ANG II clearly activated p53, which was the negative regulator of the miR17-92 cluster (Figure 4a). STAT3 and c-myc were slightly influenced, implying that they were not involved in the regulation of miR17-92 during ANG II exposure. We added the p53 inhibitor pifithrin-α (PFT) to evaluate the expression profiles of miRNAs derived from miR17-92 (Figure 4b). Based on the quantitative PCR (qPCR) results, PFT markedly restored the expression of these miRNAs, especially miR-18 (ANG II *versus* ANG II+PFT: 10.77±2.62 *versus* 60.17±15.42% *P*<0.001). HSF2 and IGF-IIR were inhibited when challenged with PFT or p53 knockdown (Figure 4c; Supplementary Figure 3E). In contrast, the overexpression of p53 clearly magnified ANG II-induced IGF-IIR and HSF2 expression (Figure 4d). Additionally, p53 overexpression severely amplified the expression levels of the ANG II-induced cardiac hypertrophy markers ANP (ANG II *versus* GFP-p53+ANG II: 1.43±0.13 *versus* 3.01±0.92-fold change) and BNP (ANG II *versus* GFP-p53+ANG II: 2.55±0.67 *versus* 7.41±1.59; *P*<0.001) (Figure 4e), which demonstrated that p53 participated in hypertension-induced cardiac hypertrophy via modulating HSF2 and IGF-IIR.

**Figure 5:**
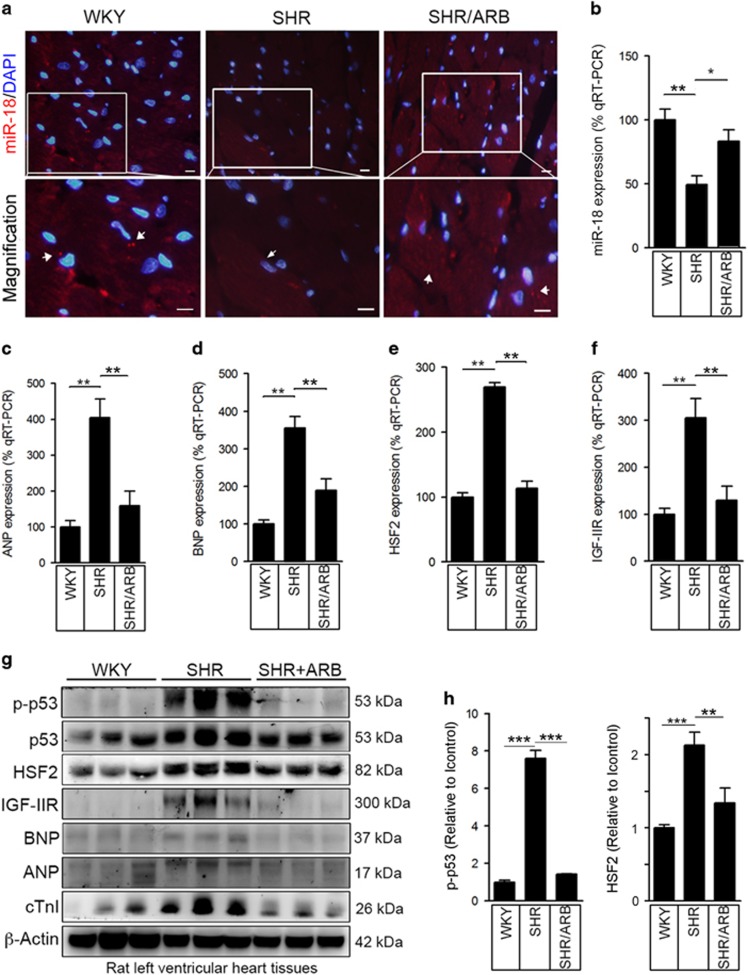
miR-18 was negatively correlated with HSF2 expression in a spontaneous hypertension-induced heart failure animal model. (**a**) The angiotensin II receptor blocker (ARB) irbesartan was consecutively administered to 16-week-old spontaneously hypertensive rats (SHRs) for 6 weeks. The animals were sacrificed and the heart tissues were isolated for further analysis. The miR-18 expression pattern was detected by fluorescence *in situ* hybridization (FISH). (**b**) The rat left ventricular heart tissues were homogenized and extracted for analysis. miR-18 expression was verified by qRT-PCR. Quantification of the results is shown (*n*=4 per group). *P<0.05 and **P<0.01. (**c**,**d**) The cardiomyopathy markers ANP and BNP were detected by qRT-PCR. Quantification of the results is shown (*n*=4 per group). **P<0.01. (**e**,**f**) HSF2 and IGF-IIR expression was detected by qRT-PCR. Quantification of the results is shown (*n*=4 per group). **P<0.01. (**g**,**h**) Rat left ventricular heart tissues were homogenized and extracted for analysis. The p53, p-p53, HSF2, IGF-IIR, ANP, BNP and cTnI expression levels were measured by western blotting analysis. Quantification of the results is shown (*n*=4 per group). **P<0.01 and ***P<0.001. Data represent means±S.D. All presented blots and micrographs are representative of three sets of independent experiments

To confirm that the transcriptional activity of p53 was indispensable for mediating HSF2 and IGF-IIR, the defective form of p53 (p53^S240R^ with only 50% p53 activity) and the dominant negative form p53^R249S^ were utilized to observe the influence of p53 on HSF2 and IGF-IIR.^28^ The immunoblotting and qPCR results showed that p53^WT^ significantly enhanced ANG II-induced HSF2 and IGF-IIR expression. In contrast, p53^S240R^ and p53^R249S^ had no influence on their expression (Figures 4f and g). Similar results were observed using a lentiviral shRNA against p53, implying that p53 was a positive regulator of ANG II-induced HSF2 and IGF-IIR upregulation (Supplementary Figure 3E). miR-18 expression was conversely lower and its target *hsf2*-3’UTR was higher when incubated with p53^WT^ compared with p53^S240R^ (Figures 4h and 5i). miR-18 expression was retained in NRVMs expressing p53^S240R^ even following exposure to ANG II (p53^WT^ *versus* p53^S240R^: 20.43±6.06 *versus* 74.00±6.76% *P*<0.01). In contrast, the fluorescent intensity of *hsf2*-3’UTR was higher in NRVMs expressing p53^S240R^ even following exposure to ANG II (p53^WT^ *versus* p53^S240R^: 166.43±6.06 *versus* 126.00±6.79% *P*<0.001). Taken together, these results revealed that p53, which was the negative regulator of miR-18, was activated to upregulate HSF2 expression, thereby contributing to IGF-IIR-induced cardiac hypertrophy.

### miR-18 mediated HSF2 expression in IGF-IIR-induced cardiac hypertrophy *in vivo*

To validate these results *in vivo*, we consecutively administered the angiotensin II receptor blocker (ARB) irbesartan to 10-week-old spontaneously hypertensive rats (SHRs) for 6 weeks. The echocardiographic analysis showed that the left ventricular fractional shortening (FS%) and ejection fraction (EF%), which are measurements of systolic heart function, were significantly decreased in the SHRs. The heart functions were almost fully rescued when the rats were administered the ARB (Supplementary Figures 4A and B). Then, we sacrificed the rats and isolated their heart tissues for analysis. Remarkably, miR-18 displayed a low density in the SHRs but was restored when the SHRs were administered the ARB based on the detection of fluorescence *in situ* hybridization (FISH) assay and the results of the quantitative RT-PCR (Figures 5a and b). Expression of the cardiomyopathy markers ANP and BNP became progressively elevated in the SHR hearts but was significantly decreased in the SHR/ARB hearts (Figures 5c and d). Furthermore, expression of HSF2 and its downstream factor IGF-IIR showed similar results (Figures 5e and f).

We surveyed the protein expression levels in the left ventricular heart tissues (Figure 5g). p53 and HSF2 were remarkably upregulated. The IGF-IIR-mediated cardiac hypertrophic markers BNP, ANP and cTnI were significantly elevated. However, ARB (irbesartan) treatment completely inhibited the expression of these proteins to suppress IGF-IIR-induced cardiac hypertrophy (Figures 5g and h). Overall, these results demonstrated that hypertension-induced p53 activation contributed to miR-18 downregulation, which in turn remarkably elevated HSF2 expression and activation. Finally, HSF2 upregulated IGF-IIR, thereby contributing to cardiac hypertrophy.

### Cardiac-specific expression of miR-18 by AAV transduction rescued hypertension-induced cardiac dysfunction

**Figure 6:**
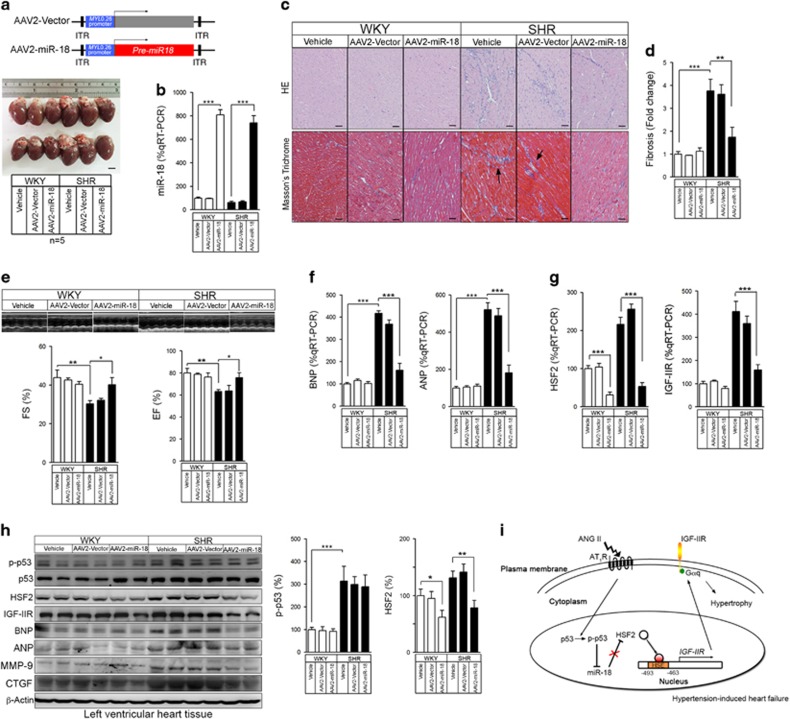
Restoration of cardiac AAV2-MLC_0.26_-miR-18 transduction improves cardiac function. (**a**) The schematic diagram of cardiac-specific AAV2-MLC_0.26_-pre-miR-18. Gross morphology of heart samples 8 weeks post-injection. Scale bars: 0.5 mm. (**b**) miR-18 expression in the hearts was detected by qRT-PCR. Quantification of the results is shown (*n*=3 per group). ***P<0.001. (**c**) HE and masson’s trichrome staining of heart samples from SHRs injected with AAV2-miR-18 and WKY rats injected with AAV2-miR-18 or the AAV2-vector control. Scale bars: 50 *μ*m. (**d**) Fibrosis of the heart samples by assessing masson’s trichrome staining. Quantification of the results is shown (*n*=4 per group). **P<0.01 and ***P<0.001. (**e**) M-mode echocardiography images and left ventricular FS and EF of SHRs injected with AAV2-miR-18 and WKY rats injected with AAV2-miR-18 or the AAV2-vector control. Quantification of the results is shown (*n*=5 per group). *P<0.05 and **P<0.01. (**f**) ANP and BNP mRNA levels in the hearts of SHRs injected with AAV2-miR-18 and WKY rats injected with AAV2-miR-18 or the AAV2-vector control. Quantification of the results is shown (*n*=3 per group). ***P<0.001. (**g**) HSF2 and IGF-IIR mRNA levels in the hearts of SHRs injected with AAV2-miR-18 and WKY rats injected with AAV2-miR-18 or the AAV2-vector control. Quantification of the results is shown (*n*=3 per group). ***P<0.001. (**h**) Immunoblotting to detect p-p53 protein expression in the hearts of SHRs injected with AAV2-miR-18 and WKY rats injected with AAV2-miR-18 or the AAV2-vector control. Quantification of the results is shown (*n*=3 per group). *P<0.05, **P<0.01 and ***P<0.001. (**i**) A working model signifying the p53-miR 18-HSF2-IGF-IIR pathway in the regulation of cardiac hypertrophy and heart failure. Values represent means±S.D. All presented blots and micrographs are representative of three sets of independent experiments

Because miR-18 has the potential to serve as a therapeutic candidate for cardiac hypertrophy, we generated AAV2 vectors to induce cardiac-specific expression of pre-miR-18 (~300 bp) under the control of the cytomegalovirus (CMV)-enhanced 260 bp myosin light chain (CMV_enh_/MLC0.26) promoter.^29^ We injected 10^12^ genomic particles of AAV2 intravenously into 15-week-old SHR rats and sacrificed the rats after 8 weeks for analysis (Supplementary Figure 5A). The miR-17 did not elevate after miR-18 injected (Supplementary Figure 5B) and these miR-18 specific expressed in heart tissue (Supplementary Figure 5C). The hearts from the AAV2-MLC-miR-18-transduced SHRs showed a well-preserved cardiomyocyte (Figures 6a–c) and reduced perivascular fibrosis compared with the hearts from the SHR animals (Figure 6d). The transduction of AAV2-MLC-miR-18 into the SHR rats resulted in better cardiac functions and pressures as assessed by echocardiography, including the FS and EF (Figure 6e). The levels of the ANP and BNP markers for cardiomyopathy and heart failure were significantly lower in the AAV2-MLC-miR-18 SHR animals compared with the AAV2-MLC-vector SHR animals based on the quantitative RT-PCR (Figure 6f). The IGF-IIR and HSF2 expression levels were also dramatically reduced in the AAV2-MLC-miR-18 SHR animals (Figure 6g). Interestingly, p53 activation was also observed in the AAV2-MLC-miR-18 SHR animals (Figure 6h). The fibrosis markers connective tissue growth factor (CTGF) and matrix metalloproeinase 9 (MMP-9) were significantly reduced in the AAV2-MLC-miR-18 SHR animals (Figure 6h). Taken together, these results suggest that rats with cardiac-specific transduction of miR-18 in the heart exhibited preserved cardiac functions and protection from hypertension-induced cardiac dysfunction.

## Discussion

Our study provided the first *in vivo* data to demonstrate that cardiomyocyte-specific overexpression of miR-18 is sufficient to protect cardiac hypertrophy and cardiac function, uncovering a new insight into the mechanism of miRNA-regulated cardiac hypertrophy. We showed that miR-18 was required for HSF2 suppression and indispensable for the regulation IGF-IIR signaling during cardiac hypertrophy and cardiac functions by maintenance of cardiomyocyte shapes for heart function. Interestingly, activation of p53 markedly downregulated the biogenesis of the miR17-92 cluster including miR-18, which resulted in HSF2 stability. HSF2 selectively activates the biogenesis of IGF-IIR for cardiac hypertrophy during hypertension-induced heart failure. Our results suggest that the modulation of the p53-miR-18-HSF2-IGF-IIR pathway in the heart is essential for cardiomyocyte morphology and cardiac function, which profoundly influence cardiac hypertrophy during hypertension-induced heart failure.

Transcriptional regulation of HSPs by HSFs in response to stresses and exposure to pharmacological agents is an evolutionarily conserved process in eukaryotic cells.^30, 31, 32^ In mammalian cells, HSF1 is the major factor that controls stress-inducible HSP expression, whereas HSF2 has been reported to be a more selective transcriptional regulator that does not control the expression of classical HSPs.^15, 32, 33^ After the discovery of two conserved HSE consensus sequences in the IGF-IIR promoter region (nt −493 to −463), we suggested that HSF2 bound to these sites to regulate IGF-IIR transcription. The results from our chromatin-IP and electrophoretic mobility shift assays confirmed that these sites in the IGF-IIR promoter were HSEs for HSF2 binding. Our previous results identified another HSE (nt −733 to −706) located on IGF-IIR that could be recognized by HSFs.^8, 34^ However, the effects of these sites on the promoters were profoundly distinct. Our results showed that HSF2 positively regulated IGF-IIR transcription, which led to cardiac hypertrophy under hypertension-induced heart failure conditions, whereas HSF1 markedly inhibited IGF-IIR transcription to protect cardiomyocytes from hypertension-induced heart failure. These findings are consistent with previous evidence that different HSFs can have targets in common^35, 36, 37^ and that the functions of HSF1 and HSF2 on promoters are different.^5, 32, 38, 39, 40, 41^ The sequence analysis results showed that the HSE on the IGF-IIR promoter recognized by HSF2 was less extensive and loosened the HSE sequence recognized by HSF1, which was similar to earlier studies that revealed that HSF2 was able to bind shorter HSEs than HSF1.^5, 38, 39^ The recognition of less extensive HSEs by HSF2 could provide an advantage when accessing HSEs in compacted chromatin regions. However, we cannot exclude the possibility that HSF1–HSF2 formed heterotrimers on the same HSE because HSF1 can directly interact with HSF2 for gene regulation.^3, 4, 5, 32^

Moreover, we found that the mechanism underlying HSF2 regulation was elegant. In further support of the hypothesis that the p53-miR-18-HSF2-IGF-IIR2 pathway controls cardiomyopathy in the heart, we noted that miR-18 transgenic mice exhibited alleviated dilated cardiomyopathy and heart failure *in vivo*. The miR17-92 cluster is the first group of miRNAs to be implicated in a developmental syndrome in humans.^42^ Several findings support a critical role for the miR17-92 cluster in the development of cardiac and smooth muscle tissues, suggesting that strict regulation of miR17-92 expression levels is required for normal cardiovascular development and function^12, 43, 44^ and protects the heart by diminishing apoptosis and alleviating ischemia/reperfusion injury.^45^ Therefore, miR17-92 might be a new regulatory target for patients with myocardial infarction. Our results indicated decreased miR-18, which in turn promoted IGF-IIR signaling to augment cardiac hypertrophy during hypertension-induced heart failure. Supporting these results, decreased miR-18a, miR19a and miR19b expression accelerated aging-induced cardiac remodeling.^10^ van Almen GC *et al.* indicated that miRNA expression of the miR-17–92 cluster changes with cardiac aging and associates decreased miR-18a, miR-19a and miR-19b expression with age-related remodeling in the heart.^10^ Moreover, miR-18 targeting of HSF2 expression was established by Bjork *et al.*, who showed that increased HSF2 transcription in spermatogenesis was controlled by miR-18.^18^ More recently, Chen *et al.*, demonstrated that the miR17-92 cluster was required and sufficient for cardiomyocyte proliferation in the adult heart,^43^ which supported our results of AAV2 cardiac-specific miR-18 gene delivery to alleviate hypertension-induced cardiomyopathy. Collectively, our results suggest that proper and balanced expression of miR-18 and HSF2 is essential for cardiac functions. These findings provide evidence for the involvement of miRNAs in the regulation of cardiac function and identify them as potential new therapeutic targets for the modulation of cardiac functions. We predict that dysregulation of these miRNAs will be associated with cardiomyopathy in human patients.

In summary, our findings revealed that the p53-miR-18-HSF2-IGF-IIR pathway controlled cardiomyopathy in the heart *in vitro* and *in vivo*, implying the therapeutic potential of miR-18 for the control of cardiac functions during hypertension-induced heart failure. Our study may shed light on the functional mechanisms of miR-18 and heart failure. Notably, investigating and understanding this regulation will enable the identification of new therapeutic targets to treat cardiovascular diseases.

## Materials and methods

### Experimental animals and oral administration of anti-hypertension drugs

All animal experiments were performed in accordance with the Guide for the Care and Use of Laboratory Animals (National Institutes of Health Publication No. 85-23, revised 1996) under a protocol approved by the Animal Research Committee of China Medical University, Taichung, Taiwan.

Spontaneously hypertensive rats (SHR) and normotensive control Wistar Kyoto rats (WKY) were used in our experiments. The rats were housed at a constant temperature (22 °C) on a 12-h light/dark cycle with food and tap water. The animals were arranged into three groups: WKY rats, SHR rats, and SHR rats treated with irbesartan (SHR/ARB). Each group contained five female 12-week old animals. The angiotensin II receptor blocker (ARB) drug irbesartan (40 mg/kg/d; Merck, Jacarepaguá, Brazil) was placed in the drinking water.

### Neonatal rat ventricular myocyte primary culture

NRVMs were prepared and cultured using a Neonatal Rat/Mouse Cardiomyocyte Isolation Kit (Cellutron Life Technology, Baltimore, MD, USA). The hearts from 1- to 3-d-old Sprague Dawley rats were dissected and transferred to a sterile beaker. Each heart was digested in the beaker with stirring at 37 °C for 12 min. The supernatant was then transferred to a new sterile tube and spun at 1200 r.p.m. for 1 min. The cell pellets were then resuspended in D3 buffer and preplated for 1 h by seeding on an uncoated plate at 37 °C in a CO_2_ incubator to select cardiac fibroblasts. The unattached cells were transferred to plates that were precoated with NS medium (supplemented with 10% fetal bovine serum). After overnight culture, the NS medium was replaced with serum-free NW medium. The cardiomyocyte cultures were ready for experiments 48 h after the initial plating.

### Expression plasmids and gene construction

Flag-HSF2 was a gift from Dr. Ying-Lei Miao (Department of Gastroenterology, the First Affiliated Hospital of Kunming Medical University, Yunnan, China). p53-GFP was a gift from Geoff Wahl (Addgene plasmid # 11770). pcDNA3.1/V5-His-TOPO-mir17-92 was a gift from Joshua Mendell (Addgene plasmid # 21109).^46^ The p53-wt, p53^S240R^ and p53^R249S^ were gifted from Jiunn-Liang Ko (Institute of Toxicology, Chung Shan Medical University, Taiwan). The IGF-IIR luciferase reporter constructs, pGL4.1-IGF-IIR-1.3 kb, pGL4.1-IGF-IIR-0.6 kb and pGL4.1-IGF-IIR-0.4 kb were generated as previously described.^17^ pGL4.1-IGF-IIR-0.6 kb-mBS1, pGL4.1-IGF-IIR-0.6 kb-mBS2 and pGL4.1-IGF-IIR-0.6 kb-mBS1/2 were generated by the QuickChange II site-direct mutagenesis kit (Agilent Technologies, Santa Clara, CA, USA). The pLKO.1 plasmids expressing shRNA against p53, HSF2 and IGF-IIR were purchased from Sinica (Taiwan, ROC).

The cells were grown to 80% confluence on the day of transfection. Briefly, the siRNA and plasmids were transfected into H9c2 and NRVMs cells using PureFection (LV750A-1, System Biosciences, CA, USA).

### Cardiac-specific AAV2 vectors generation and recombinant AAV2 virus purification

Human pre-miR-18 was generated by PCR (5'AAV-miR-18-XbaI: 5′-ATCTCTAGAAAATTTAGCAGGAAAAAAGAGAACAT-3′ 3'AAV-miR-18-HindIII: 5′-GGCAAGCTTACAATAAAAGTACACAA AATTAGT-3′) and subcloned into a pAAV2ss-CMV-MLC260 vector, which contained one cardiac-specific promoter myosin light chain 260 promoter (SB-P-AA-003-01, SIRION Biotech, Martinsried, Germany).

The viruses were all produced by the triple transfection method, MLC260-pre-miR-18 and the helper plasmids pCR2-miR342 and pHelper, using 293T cells. Seventy-two hours after transfection, the cells were collected by centrifugation and recombinant AAV2 vectors were produced and purified using AAVpro purification kit (6232, Takara, Japan). AAV2 Titration was performed by quantitative PCR (qPCR) on vector genomes.

### *In vivo* gene transfer

All procedures were approved by the local animal care committee. All procedures involving the use and care of animals were performed according to the Guide for the Care and Use of Laboratory Animals published by the National Research Council (NIH Publication No. 85-23, revised 1996). 15-week-old female WKY and SHR rats (200–250 g) were administered 10^12^/0.5 ml viral genomes of AAV2 *via* tail vein injection, starting with *n*=6 per group. Control animals were injected with 0.5 ml phosphate-buffered saline supplemented with 5% sorbitol. The injections into the veins were carried out using 28 Gauge needles. All the rats recovered from the injection quickly without loss of mobility or interruption of grooming activity. Eight weeks post-injection, animals were euthanized and various organs (heart, skeletal muscle, lung, liver, spleen and kidney) were harvested for analysis.

### Construction of miR-18 GFP reporters

A construct containing 3′UTR of HSF2 was made by amplifying from mRNA region (350 base pairs) by the primers as followed: 5'HSF2-UTR(1)-KpnI 5′-AGTGG TACCATCCCCAGGAAGTGGACTTTAC-3′and 3′HSF2-UTR(350)-BamHI 5′-ACAGGATCCGCAAATAGACAGCATCA AACAG-3′. Then, the 3′UTR of HSF2 miR-18 targeting region was subcloned into pEGFP-C1 containing a *gfp* gene between KpnI and BamHI restriction sites.

The mutation on miR-18-targeting site of 3’UTR of HSF2 was generated by the QuikChange II site-direct mutagenesis kit (Agilent Technologies, CA, USA). These primers used as followed: 5'HSF2-UTR-mutant: 5′-GTTAGTGAGAAAAGCAAAAAGGGTTTTTATTCCACAGTATCTGATTAAACAAAACAAAGC-3′ 3′HSF2-UTR-mutant: 5′-GCTTTGTTTTGTTTAATCAGATACTGTGGAAT AAAAACCCTTTTTGCTTTTCTCACTAAC-3′.

### RNA extraction, RT-PCR and qRT-PCR

Total RNA was extracted using the Direct-zol RNA MiniPrep Kit (Zymo Research Corporation, Irvine, CA, USA) according the manufacturer’s instructions. Briefly, 1 *μ*g of total RNA was incubated with 0.5 *μ*g of oligo dT (MD.Bio., Taipei, Taiwan) at 70 °C for 15 min. Then, the RNA was mixed with buffer containing 0.25 mM dNTPs (MD. Bio., Taipei, Taiwan), 20 U of RNasin I Plus RNase Inhibitor (Promega, WI, USA) and 20 U of M-MLV Reverse Transcriptase (Promega) and incubated at 42 °C for 90 min for cDNA synthesis. This mixture was then used for specific cDNA amplification in a GeneAmp PCR system 2400 (Perkin Elmer, Waltham, MA, USA).

For mRNA quantification, Real-time PCR was performed using a standard LightCycler 480 SYBR Green I Master protocol on an LightCycler 96 System Roche, Basel, Switzerland). The 10 *μ*l PCR included 2 *μ*l RT product, 5 *μ*l 2 × SyberGreen PCR Mix, 0.5 *μ*l 10 *μ*M forward primer, 0.5 *μ*l 10 *μ*M reverse primer and 2 *μ*l ddH_2_O. All reactions were run in triplicate. The cycle number at which the reaction crossed the threshold cycle (Ct) was determined for each gene and the relative amount of each gene to GAPDH was described using the equation 2^ΔCt^ where ΔCt=(Ct_interested gene_ – Ct_GAPDH_).

### miRNA detection by qRT-PCR

Detection of the mature miRNAs was performed by reverse transcription using the miRNA cDNA synthesis kit (Takara, Shiga, Japan) according to the manufacturer's instructions, and the RT-qPCR reaction was performed using a standard LightCycler 480 SYBR Green I Master protocol on an LightCycler 96 System (Roche, Basel, Switzerland). The 10 *μ*l PCR included 2 *μ*l RT product, 5 *μ*l 2 × SyberGreen PCR Mix, 0.5 *μ*l 10 *μ*M forward primer, 0.5 *μ*l 10 *μ*M reverse primer and 2 *μ*l ddH_2_O. The reactions were incubated in a 32-well plate at 95 °C for 10 min, followed by 40 cycles of 95 °C for 15 s, 55 °C for 15 s and 72 °C for 30 s. All reactions were run in triplicate. The cycle number at which the reaction crossed the threshold cycle (C_t_) was determined for each gene and the relative amount of each miRNA to U6 rRNA was described using the equation 2^ΔCt^ where ΔC_t_=(C_t_miRNA – C_t_U6 rRNA). The primer we used to detect miRNA expression as lists: Rho-miR17-5p (5′-CAAAGTGCTTACAGTGCAGGT AG-3′), Rho-miR-18-5p (5′-CTATCTGCACTAGATGCACCTTA-3′), Rho-miR19a-3p (5′-TGTGCAAATCTATGCAAAACTGA-3′), Rho-miR20a-5p (5′-TAAAGTGCTTATAGTGCAGGTAG-3′), Rho-mir-92a-1-3p (5′-TATTGCACTTGTCCCGGCCTG-3′).

### miRNA *in situ* hybridization analysis

To prepare the probes we used the following synthesized oligonucleotides, which sequences are complementary to each 21 nucleotide mature miRNA of interest, rho-miR-18a-5p: 5′-TAAGGT GCA TCT AGT GCA GAT A-3′. Locked nucleic acid (LNA)–modified oligonucleotide probes labeled with FITC at their 3′-ends were obtained from Molecular Biology of ThermoElectron GmbH. Five-micrometer-thin sections of FFPE rat heart tissues adhered to glass slides were deparaffinized in three consecutive xylene baths for 1 min each, followed by 1 min each in serial dilutions of ethanol (100%, 100%, 95%, 95%). Slides were incubated with 100 *μ*g/ml Protease K (in 50 mM Tris pH=7.5) at 25 °C) for 30 min, washed thrice with ddH_2_O, submerged in 75% ethanol for 1 min, and air-dried completely. Slides were then hybridized in incubation chambers at 75 °C for 5 min, using 1 *μ*g/ml LNA–modified probes diluted with Hybridization buffer (6 × SSC, 10% Goat Serum, 50% Formamide), and then incubated at 37 °C overnight in an oven. After hybridization, slides were rinsed thrice in 0.5 × SSC, washed for 30 min at 50 °C in 0.5 × SSC, and rinsed twice in TBS. Nucleus are labeled with DAPI.

### miR-18 mimic and antimir transfection

Chemically modified sense RNA (rho-miR-18 mimic) or antisense RNA (rho-miR-18 antimir) was synthesized by (Qiagen, Hilden, Germany). Transfection with the miR-18 mimic or antimir was performed using PureFection Reagent (System Biosciences, Palo Alto, CA, USA). Briefly, 10 nM of miR-18 mimic or antimir was mixed with 20 *μ*l PureFection in 100 *μ*l serum-free culture medium for 10 min at room temperature to form transfection complexes. The cells were incubated with the transfection complexes for 48 h.

### Cell culture and transient transfection

H9c2 cardiomyoblast cells derived from embryonic BD1X rat heart tissue were obtained from the American Type Culture Collection (ATCC, Manassas, VA, USA) and cultured in Dulbecco’s modified essential medium supplemented with 10% fetal bovine serum, 2 mM glutamine,100 U/ml penicillin, 100 mg/ml streptomycin and 1 mM pyruvate in humidified air (5% CO_2_) at 37 °C.

The cells were grown to 80% confluence on the day of transfection. Plasmids and siRNAs were transfected using the PureFection transfection reagent according to the manufacturer’s instructions (System Biosciences). All siRNAs were purchased from Sigma (St. Louis, MO, USA).

### Antibodies and reagents

The following antibodies were used in this study: anti-IGF-IIR (#ab124767, Abcam, Cambridge, UK), anti-HSF2 (#sc-13056, Santa Cruz, CA, USA), anti-p53 (#2524, Cell Signaling Technology, MA, USA), anti-phospho-p53 (#9284, Cell Signaling Technology, Danvers, MA, USA), anti-BNP (sc-18818, Santa Cruz), anti-ANP (sc-20158, Santa Cruz), anti-*β*-actin (sc-47778, Santa Cruz), anti-cTnI (ab19615, Abcam), anti-HDAC1, anti-GAPDH (sc-137179, Santa Cruz), anti-STAT3 (sc-483, Santa Cruz), anti-c-myc (sc-42, Santa Cruz), anti-Tubulin (sc-5286, Santa Cruz) and anti-phospho-cTnI (#4004, Cell Signaling Technology). All secondary antibodies (HRP-conjugated anti-rabbit, anti-mouse and anti-goat) were purchased from Santa Cruz Biotechnology. All reagents were purchased from Sigma.

### Luciferase and fluorescence reporter assay

Briefly, cells were co-transfected with both luciferase reporter constructs and internal control luciferase plasmids. After transfection and treatment, the cells were assayed for luciferase activity using a Dual-Glo luciferase assay system (Promega). Plates were read on a Reporter Microplate Luminometer (Turner Biosystems, Sunnyvale, CA, USA). To control for potential variations in transfection or lysis efficiency, the luciferase signals were normalized to the internal control luciferase signal.

The HSF2-3′UTR GFP reporters were transfected into cell, and then assayed for the GFP activity by Reporter Microplate Luminometer (Turner Biosystems). The GFP signals were normalized to the GFP control signal.

### Western blot analysis and immunoprecipitation

For these analyses, 30 *μ*g of the total lysates or 10 *μ*g of the subcellular fractions was separated through 6–12% SDS-PAGE, then transferred to a PVDF membrane (GE, Amersham, UK). The membranes were blocked using 5% non-fat milk and blotted with specific antibodies overnight at 4 °C. Then, the protein signals were measured using horseradish peroxidase-conjugated secondary antibodies (1:10 000, GE Healthcare, Amersham, UK) and the Immobilon Western Chemiluminescent HRP Substrate (Milliore, Billerica, MA, USA). Densitometric analysis of the immunoblots was performed with the AlphaImager2200 digital imaging system (Digital Imaging System, San Diego, CA, USA). The digital images were processed in Adobe Photoshop 7.0. Each blot was stripped using Restore Western Blot Stripping Buffer (Pierce, Rockford, IO, USA) and incubated with other antibodies.^47, 48^ The results were analyzed and quantified using Image J software (NIH, Bethesda, MD, USA).

Immunoprecipitation was performed from H9c2 cell lysates using the PureProteome Protein G Magnetic Bead System (Millipore) according to the manufacturer’s instructions.^48^ First, 300 *μ*g of the cell lysate was prepared. The lysate was then combined and allowed to interact with 2 *μ*g of a specific primary antibody, and the mixture was incubated on a rotator at 4 °C overnight. Immunoprecipitated proteins were eluted from the magnetic beads at 95 °C for 5 min and separated by SDS-PAGE. The proteins were transferred to a PVDF membrane and probed with specific antibodies.

### Indirect immunofluorescence and confocal microscopy

Cells were fixed with 4% paraformaldehyde for 15 min at room temperature and permeabilized with 0.1% Triton X-100 for 15 min at room temperature before staining with a specific antibody.^47^ Then, the cells were washed and stained with Alexa 546 rabbit anti-mouse IgG secondary antibodies (Invitrogen, Carlsbad, CA, USA). Images were captured using a Leica SP2 Confocal Spectral Microscope. The images were processed using Adobe Photoshop.

### Nuclear extraction

H9c2 cells and NRVMs grown in 10-cm cell culture dishes were harvested by scraping in 1 ml of 1 × ice-cold PBS after treatment. Nuclear fractions were prepared in a cytosol extraction buffer (CEB) containing 1 mM DTT and protease inhibitors, then separated from cytosolic fractions by a nuclear extraction buffer (NEB) supplemented with 1 mM DTT and protease inhibitors using the Nuclear/Cytosol Fractionation Kit (BioVision, Milpitas, CA, USA) according to the manufacturer’s direction. The nuclear fractions were quantified by Bradford assays (Bio-Rad) and 30 *μ*g of extracted nuclear proteins was separated on 8% SDS-PAGE for western blot analysis.

### Chromatin immunoprecipitation assay

A ChIP assay was performed using lysates from H9c2 cells that were treated with ANG II. The assay was performed using EZ ChIP Chromatin (Millipore) according to the manufacturer’s instructions. The cells were washed in PBS three times and incubated for 10 min with 1% formaldehyde. After quenching with 0.1 M glycine, the cross-linked material was sonicated into chromatin fragments with an average length of 500–800 bp. The chromatin was kept at −80 °C. The chromatin solution (100 *μ*l of chromatin sample and 900 *μ*l of dilution buffer) was pre-cleared by adding Protein G agarose for 2 h at 4 °C, and immunoprecipitation was then performed with Protein G agarose and 1–10 *μ*g of the indicated antibodies overnight at 4 °C on a rotating wheel. The immunoprecipitated material was washed five times with cold washing buffer. The crosslinks were reversed by incubating the samples with 8 *μ*l of 5 M NaCl for 5 h at 65 °C, and 10 *μ*g of RNase A was added to eliminate the RNA. The recovered material was treated with proteinase K, placed in spin columns, and precipitated. The pellets were resuspended in 50 *μ*l of double-distilled water and analyzed using PCR.

### Electrophoretic mobility shift assay

Cells were harvested and nuclear extracts were prepared using NE-PER nuclear and cytoplasmic reagents (Pierce). A mixture containing 5 *μ*g nuclear extract and 3 *μ*g poly dIdC in binding buffer (20 mM HEPES pH 7.9, 100 mM KCl, 1 mM EDTA, 1 mM DTT, 4% (v/v) Ficoll, 1 × PhosSTOP (Roche)) was incubated for 20 min on ice. DNA–protein complexes were separated on a pre-run 4% polyacrylamide gel in 0.5 × TBE with recirculation of the buffer. The gel was dried and signals were visualized using a PhosphorImager.

### Assessment of cardiomyocyte size *in vitro*

Neonatal rat ventricular cardiac myocytes were grown on slides for 24 h. After 24 h attachment, cells were treated with ANG II for 24 h and then fixed with 4% paraformaldehyde and stained for the rhodamine phalloidin (1:50 dilution, Molecular Probes, Invitrogen, Carlsbad, CA, USA). Images were analyzed to determine cell surface area. Cell images from at least ten randomly chosen fields (× 40 objective) of 60 cardiomyocytes were measured in three separate experiments using NIH image software.

### Statistical analysis

All experiments were performed at least three times. Statistical analysis was performed using GraphPad Prism5 statistical software (San Diego, CA). Statistical significance was set at *P*<0.05. Multiple comparisons of the data were analyzed through ANOVA assays. Tukey's Honestly Significant Difference tests (Tukey *HSD*) for post-hoc comparison were used with a significance level of 5%. All results were quantified using Image J (NIH, MA, USA) and processed using Adobe Photoshop.
