# Supplementary material for: p53-mediated miR-18 repression activates HSF2 for IGF-IIR-dependent myocyte hypertrophy in hypertension-induced heart failure
Source: Cell Death Dis. 2017 Aug 10;8(8):e2990–. doi: 10.1038/cddis.2017.320 (PMC5596536; doi:10.1038/cddis.2017.320)
Supplement: Supplementary Figures [file cddis2017320x1.doc]

**Supplementary Figures**

**Supplementary Figure 1**

**
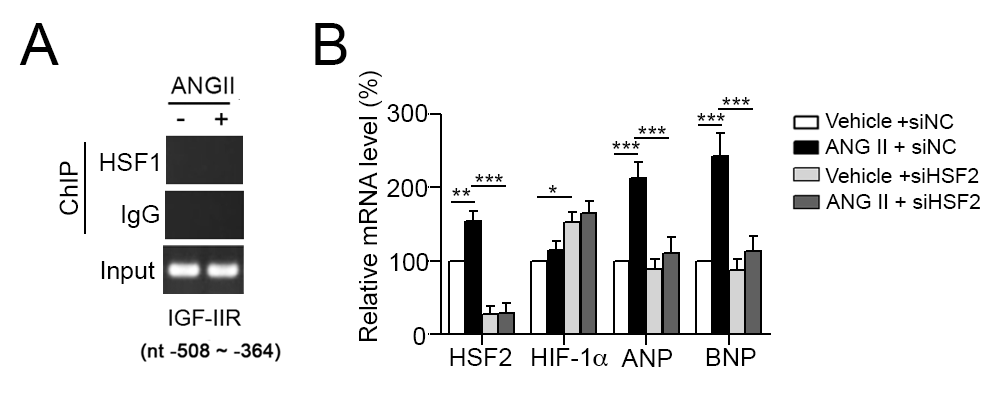
**

**Figure 1. HSF1 did not bind to the IGF-IIR promoter (nt -508 to -364).**

(A) After treatment with ANG II, the NRVMs were lysed and analyzed by chromatin immunoprecipitation (ChIP). The HSF1 binding to the IGF-IIR promoter (nt -493 to -463) was tested using PCR. (B) NRVMs were transfected with siRNA for 24 hrs, and then treated with 100nM ANG II for 24 hrs. The intensity of HSF2, HIF-1α, ANP and BNP were evaluated by qRT-PCR. **P<0.01 and ***P<0.001 represents a significant decrease.

**Supplementary Figure 2**


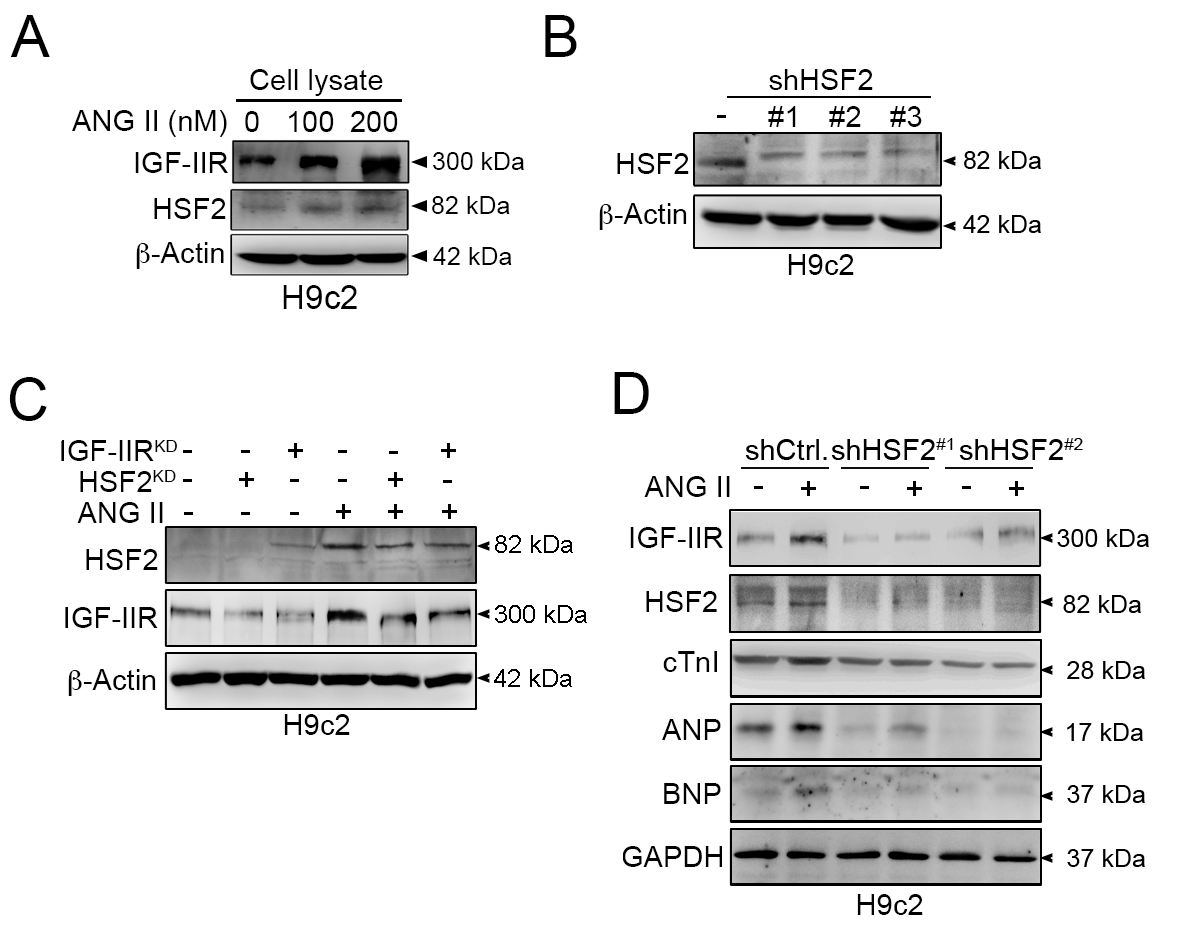


**Figure 2. HSF2 knockdown alleviated ANG II-induced hypertrophy.**

(A) H9c2 cells were treated with diverse concentration of ANG II for 24 hrs. (B) H9c2 were infected with 3 individual lentivirus carrying HSF2 shRNA and selected by puromycin. (C) H9c2, H9c2-HSF2^KD^ and H9c2-IGF-IIR^KD^ were challenged with 100 nM ANG II for 24 hrs. (D) H9c2, H9c2-HSF2^KD^ were challenged with 100 nM ANG II for 24 hrs. The HSF2, IGF-IIR and hypertrophy markers expression was measured by immunoblotting.

**Supplementary Figure 3**


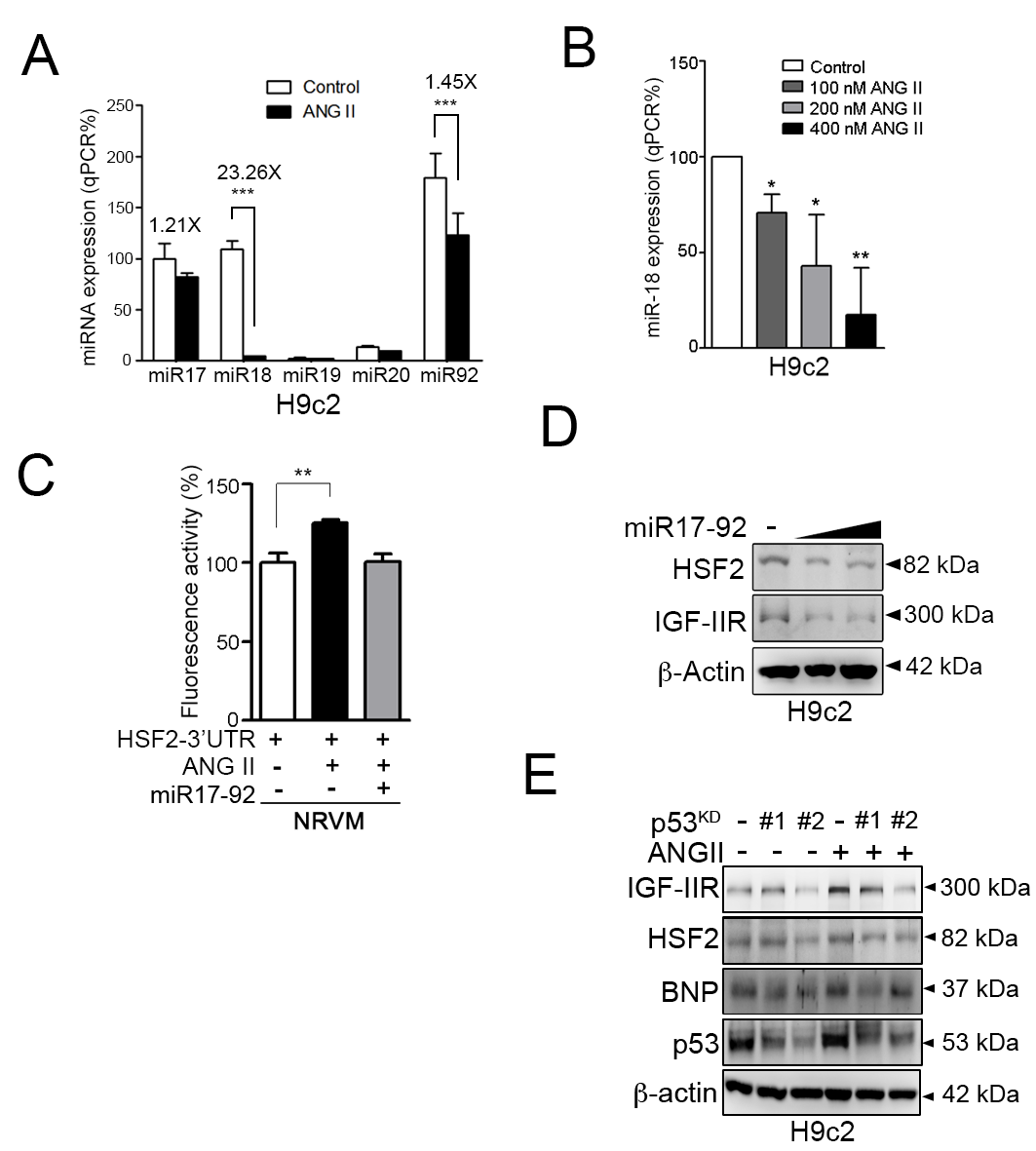


**Figure 3. miR18 expression was downregulated in ANG II-treated cardiomyocytes.**

(A) H9c2 cells were treated with 100nM ANG II for 24 hrs. The individual miRNA in miR 17-92 clusters was evaluated by qRT-PCR. ***P<0.001 represents a significant decrease. (B) H9c2 cells were treated with diverse concentration of ANG II for 24 hrs, and the level of miR18 was measured by by qRT-PCR. *P<0.05 and **P<0.01 represents a significant decrease. (C) NRVMs were transfected with HSF2-3’UTR GFP reporter plasmid and pcDNA3-miR17-92 clusters for 24 hrs, and then treated with 100nM ANG II for 24 hrs. The GFP intensity was evaluated. **P<0.001 represents a significant decrease. (D) H9c2 cells were transfected with pcDNA3-miR17-92 clusters for 48 hrs. The HSF2 and IGF-IIR expression was measured by immunoblotting. (E) H9c2-p53^KD^ cells were challenged with 100 nM ANG II for 24 hrs. The HSF2, IGF-IIR and hypertrophy markers expression was measured by immunoblotting.

Data represent means ± SD. All presented blots and micrographs are representative of three sets of independent experiments.

**Supplementary Figure 4**


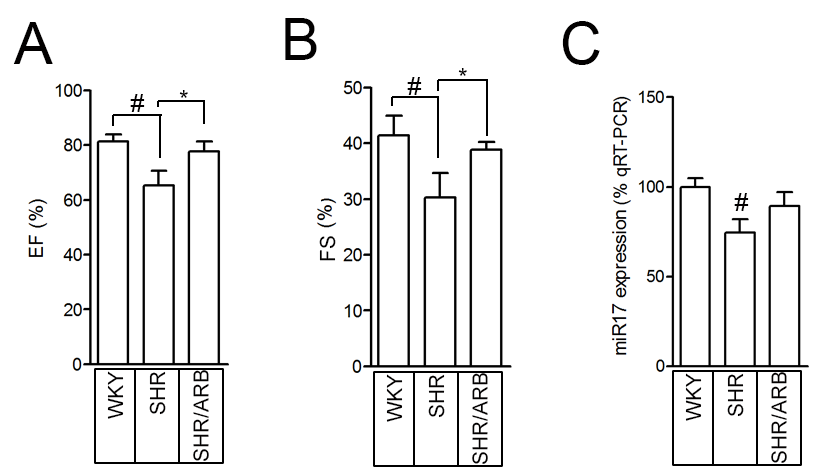


**Figure 4. The angiotensin II receptor blocker (ARB) rescued cardiac functions of spontaneous hypertensive rats (SHR)**

(A-B) The cardiac functions (EF% and FS%) were assessed by echocardiography. #P<0.05 represents a significant decrease and *P<0.05 represents a significant increase (n=4).

(C) The rat left ventricular heart tissues were homogenized and extracted for analysis. miR-17 expression was verified by qRT-PCR. ^#^P<0.05 represents a significant decrease (n=4).

**Supplementary Figure 5**


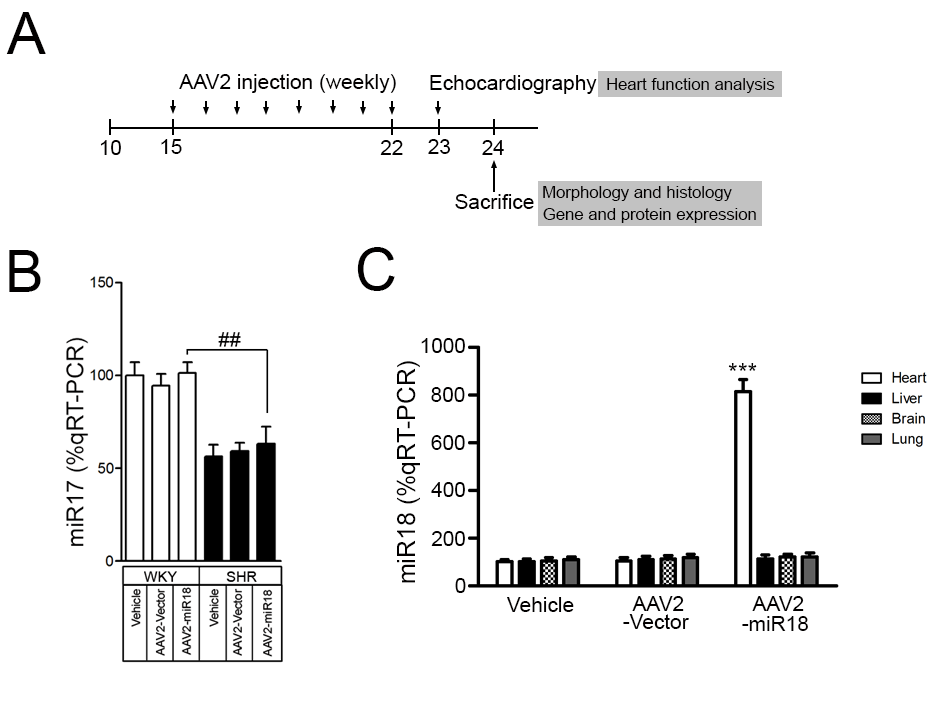


**Figure 5. Cardiac-specific AAV2-MLC_0.26_-pre-miR-18 gene delivery animal model.**

(A) Timeline of the cardiac-specific AAV2-MLC_0.26_-pre-miR-18 gene delivery.

(B) The rat left ventricular heart tissues were homogenized and extracted for analysis. miR-17 expression was verified by qRT-PCR. ^##^P<0.01 represents a significant decrease (n=4). (C) The AAV2-miR18 were specific expressed in heart tissue (n=3). ***P<0.001
